# Servindo a Dois Mestres: Resolvendo o Dilema entre Metanálise de Dados Individuais e Metanálise de Dados Agregados de Estudos com Estatinas

**DOI:** 10.36660/abc.20220539

**Published:** 2023-03-24

**Authors:** Lucas Tramujas, Fernando Azevedo Medrado, Alexandre Biasi Cavalcanti, Carlos Eduardo Pompilio

**Affiliations:** 1 Instituto de Pesquisas Hcor São Paulo SP Brasil Instituto de Pesquisas, Hcor, São Paulo, SP – Brasil; 2 Hospital das Clínicas Faculdade de Medicina Universidade de São Paulo São Paulo SP Brasil Clínica Médica, Hospital das Clínicas, Faculdade de Medicina da Universidade de São Paulo (HCFMUSP), São Paulo, SP – Brasil

**Keywords:** Metanálise, Colesterol, Doença cardiovascular

## Introdução

Níveis elevados de colesterol de lipoproteína de baixa densidade (LDL-C) foram estabelecidos como um importante fator de risco para o desenvolvimento de aterosclerose.^1^ Como corolário desse raciocínio, surgiu uma abordagem centrada no LDL com base em estatinas e outros medicamentos capazes de reduzir o LDL-C e o risco cardiovascular.^1^

A *Cholesterol Treatment Trialists’ Collaboration* (Colaboração de Investigadores Clínicos no Tratamento de Colesterol), uma metanálise de dados individuais do paciente, encontrou uma relação logarítmica entre o grau de redução de LDL-C e eventos cardiovasculares.^2^ De acordo com isso, diretrizes especializadas para prevenir doenças cardiovasculares recomendam o uso de estatinas para prevenção primária e secundária.^2^

Por outro lado, uma metanálise de dados agregados publicada por Byrne et al. sobre a associação entre as reduções induzidas por estatinas nos níveis de LDL-C e as reduções absolutas e relativas em desfechos clínicos individuais mostrou resultados discrepantes.^3^ Os autores concluíram que as reduções de risco absoluto do tratamento com estatinas na mortalidade por todas as causas, infarto do miocárdio, e acidente vascular cerebral são modestos. A metanálise também sugere uma associação inconclusiva entre as reduções induzidas por estatina nos níveis de LDL-C e os resultados clínicos.^3^

Aqui, descreveremos as principais diferenças entre metanálise de dados individuais de pacientes e metanálise de dados agregados e como entender esses resultados para evitar viés na tomada de decisões médicas, considerando o exemplo de metanálises de ensaios com estatinas.

## Definições de metanálise de dados agregados e metanálise de dados individuais de pacientes

A metanálise de estudos de intervenção é um método estatístico para combinar todas as pesquisas publicadas sobre uma questão de pesquisa específica e estimar os efeitos gerais do tratamento agrupados.^4^

Revisões sistemáticas tradicionais e metanálises são baseadas em dados publicados agregados, o que significa que não são necessários bancos de dados de estudos originais com dados individuais dos pacientes.^4^ As medidas de efeito mais comuns usadas como uma estatística resumida são odds ratio (razão de chances), risco relativo ou diferença de risco para comparar resultados dicotômicos (por exemplo, doença versus ausência de doença) ou diferença média ou diferença média padronizada para comparar resultados contínuos (por exemplo, medição dos níveis de LDL-C) entre os grupos de tratamento.^4^

Por outro lado, a metanálise de dados individuais de participantes envolve obter, combinar e analisar bancos de dados de estudos originais.^4^ O conceito de dados individuais de paciente é diferente de dados agregados, pois o primeiro se refere aos dados coletados para cada participante do estudo, enquanto o segundo se refere às informações médias ou estimadas de todos os indivíduos em um estudo.^5^ Por exemplo, em um ensaio clínico de colesterol, os dados individuais do paciente podem ser os níveis de colesterol pré-tratamento e pós-tratamento ou características clínicas basais. Isso permitiria estimar a diferença no LDL-C para cada paciente e avaliar o tamanho da associação dessas diferenças no LDL-C e os desfechos clínicos. A Tabela 1 lista as vantagens e desvantagens dos dados agregados e da metanálise de dados individuais de pacientes.

**Tabela 1:** – Resumo das vantagens e desvantagens da metanálise de dados agregados e da metanálise de dados individuais de pacientes

	Vantagens	Desvantagens
**Metanálise de dados agregados**	Mais fácil de executar	Depende da qualidade dos dados originais e de como os autores os relatam
	Requer menos tempo	Os desfechos primários e secundários do estudo podem diferir daqueles examinados na metanálise
	Menos caro	Achados negativos são mais propensos a serem negligenciados devido ao viés de publicação
		Poder limitado para analisar os efeitos do tratamento em subgrupos específicos de interesse que diferem daqueles avaliados em estudos primários
**Metanálise de dados individuais de pacientes**	Dados preliminares e não publicados podem ser incluídos, reduzindo o risco de viés de publicação	Demorado devido à aquisição de dados, extração, análise e necessidade de colaboração
	Avaliação abrangente do protocolo, métodos e qualidade geral do estudo	A coleta de dados de certos estudos, particularmente os mais antigos, pode ser difícil
	Permite a análise de subgrupo para entender melhor o impacto de uma intervenção no nível do paciente	Mais caro
	Se aplicável, a análise estatística pode ser ajustada às diferenças da linha de base	Mais experiência necessária

## Quais são as diferenças entre a metanálise de dados individuais do paciente e a metanálise de dados agregados dos ensaios com estatinas em termos de resultados?

A *Cholesterol Treatment Trialists’ Collaboration* , uma metanálise de dados individuais de pacientes com 170.000 pacientes e 26 estudos clínicos, teve como objetivo avaliar a segurança e eficácia de reduções mais intensivas de LDL-C.^2^ Dados individuais de participantes de ensaios clínicos randomizados comparando tratamentos mais intensivos versus regimes de estatina menos intensivos (5 ensaios; 39.612 indivíduos) e estatinas versus placebo foram incluídos (21 ensaios; 129.526 indivíduos). Eles mostraram uma redução nos principais desfechos cardiovasculares para cada redução de 1 mmol/L no LDL-C (razão de taxas, 0,78; intervalo de confiança [IC] de 95%, 0,76 a 0,80; p<0,0001).^2^

A metanálise de dados agregados publicada por Byrne et al. para avaliar uma questão semelhante obteve resultados inconclusivos.^4^ A associação entre redução do LDL-C e infarto do miocárdio não foi estatisticamente significativa, a associação entre redução do LDL-C e óbito foi significativa apenas na escala relativa, mas não absoluta, e a associação entre a redução do LDL-C e acidente vascular cerebral foi significativa com uma redução nos riscos absolutos e relativos.^3^ Este estudo usou meta-regressão, que analisa a redução agregada no LDL-C e o tamanho do efeito (agregado) de cada ensaio. Por outro lado, nos dados individuais dos participantes, a metanálise considera a variação no LDL-C e o resultado de cada paciente, com uma granularidade muito maior de informações.^3^

## Como entender esses resultados discrepantes? Além disso, em que situações se espera que a metanálise de dados agregados e a metanálise de um paciente individual gerem respostas idênticas para uma pergunta específica?

Quando a questão diz respeito ao efeito comparativo de dois tratamentos alternativos em um resultado clínico em uma determinada população, a estimativa combinada do efeito dos dados agregados ou da metanálise de dados de pacientes individuais deve ser semelhante.^5^

No entanto, discrepâncias podem surgir por vários motivos. Em primeiro lugar, pode haver diferenças nos estudos originais inscritos: regimes de estatinas de alta intensidade versus regimes de baixa intensidade foram considerados apenas na metanálise de dados de pacientes individuais. Como resultado, os pacientes inscritos na metanálise de dados agregados apresentavam menor risco cardiovascular. Essa característica da metanálise de dados agregados de LDL-C ajuda a explicar tamanhos de efeito absolutos menores, que dependem do risco basal (assumindo redução constante do risco relativo em diferentes riscos basais).

Em segundo lugar, a metanálise de dados agregados tem um poder menor para detectar relações entre características no nível do paciente e tamanhos de efeito. Um estudo meta-epidemiológico mostra que as estimativas de meta-regressão de dados agregados são menos precisas do que aquelas das metanálises de dados individuais de pacientes.^6^ Erros aleatórios em estimativas de meta-regressão frequentemente farão com que difiram substancialmente daqueles obtidos em metanálise de dados individuais.^7^ Essa falácia é conhecida como viés de agregação, que é definido pela suposição de que uma associação entre duas variáveis em nível de grupo é igual à associação entre a variável correspondente em nível individual.^7^

Em terceiro lugar, nem todas as características que podem impactar significativamente o funcionamento de uma intervenção podem ser adequadamente avaliadas em uma metanálise de dados agregados, mais comumente porque os efeitos de acordo com as características de linha de base de interesse não estão disponíveis nos artigos relatados.^7^

Em quarto lugar, no caso das metanálises do LDL-C, a metanálise dos dados individuais do paciente avaliou a associação entre a variação do LDL-C e um desfecho composto (morte coronariana ou infarto do miocárdio não fatal). Em contraste, a metanálise de dados agregados avaliou a associação entre LDL-C e morte, acidente vascular cerebral ou infarto do miocárdio separadamente.^2 , 3^

À medida que o número de eventos para um resultado composto é maior, a precisão das estimativas de efeito aumenta. Quando os estudos originais não relatam os efeitos do tratamento para o resultado combinado de interesse, a metanálise usando dados agregados não é possível. Por outro lado, é possível gerar e analisar resultados combinados avaliando bancos de dados de ensaios originais.

Portanto, em comparação com metanálises de dados agregados, metanálises de dados individuais são superiores na investigação de relações entre efeitos em variáveis intermediárias e desfechos clínicos e na análise de possíveis modificadores de tratamento. Por outro lado, uma metanálise de dados agregados ou individuais de pacientes deve oferecer respostas semelhantes para questões de efeito do tratamento, desde que use as mesmas definições para população, tratamento, comparador e desfechos e inclua os mesmos estudos originais.
