# Supplementary material for: Servindo a Dois Mestres: Resolvendo o Dilema entre Metanálise de Dados Individuais e Metanálise de Dados Agregados de Estudos com Estatinas
Source: Arq Bras Cardiol. 2023 Mar 24;120(4):e20220539. [Article in Portuguese] doi: 10.36660/abc.20220539 (PMC10263432; doi:10.36660/abc.20220539)
Supplement: Translation

# Translation: Serving Two Masters: Resolving the Dilemma between Individual Patient Data Meta-Analysis and Aggregate Data Meta-Analysis from Statin Trials

**Authors:** Lucas Tramujas, Fernando Azevedo Medrado, Alexandre Biasi Cavalcanti, Carlos Eduardo Pompilio

**Affiliations:** 1 Brazil Instituto de Pesquisas, Hcor, São Paulo, SP – Brazil; 2 Brazil Clínica Médica, Hospital das Clínicas, Faculdade de Medicina da Universidade de São Paulo (HCFMUSP), São Paulo, SP – Brazil

**Keywords:** Meta-Analysis, Cholesterol, Cardiovascular Disease

## Introduction

Elevated levels of low-density lipoprotein-cholesterol (LDL-C) have been established as an important risk factor for the development of atherosclerosis.^1^As a corollary of this reasoning, an LDL-centric approach has emerged based on statins and other medications able to reduce LDL-C and cardiovascular risk.^1^

The Cholesterol Treatment Trialists’ Collaboration, an individual patient data meta-analysis, found a log-linear relationship between the degree of LDL-C reduction and cardiovascular events.^2^According to this, expert guidelines for preventing cardiovascular diseases recommend statin use for primary and secondary prevention.^2^

On the other hand, an aggregate data meta-analysis published by Byrne et al. on the association between statin-induced reductions in LDL-C levels and the absolute and relative reductions in individual clinical outcomes has shown discrepant results.^3^The authors concluded that the absolute risk reductions of treatment with statins in all-cause mortality, myocardial infarction, and stroke are modest. The meta-analysis also suggests an inconclusive association between statin-induced reductions in LDL-C levels and clinical outcomes.^3^

Here, we will describe the main differences between individual patient data and aggregate data meta-analysis and how to make sense of these results to avoid bias in medical decision-making, considering the example of meta-analyses of statin trials.

## Definitions of meta-analysis of aggregate data and meta-analysis of individual patient data

Meta-analysis of interventional studies is a statistical method to combine all published research on a specific research question and estimate pooled overall treatment effects.^4^

Traditional systematic reviews and meta-analyses are based on aggregate published data, meaning that databases from original trials with data from individual patients are not needed.^4^The most common measures of effect used as a summary statistic are odds ratio, relative risk, or risk difference to compare dichotomous outcomes (e.g., disease versus no disease) or mean difference or standardized mean difference to compare continuous outcomes (e.g., LDL-C levels measurement) between the treatment groups.^4^

Conversely, the individual participant data meta-analysis involves obtaining, combining, and analyzing databases from original studies.^4^The concept of individual patient data is distinct from aggregate data in that the first refers to data collected for each study participant, while the second refers to information averaged or estimated across all individuals in a study.^5^For example, in a cholesterol trial, the individual patient data could be the pre-treatment and post-treatment cholesterol levels or baseline clinical characteristics. This would allow estimation of the difference in LDL-C for each patient and assess the size of the association of these LDL-C differences and clinical outcomes. Table 1 lists the advantages and drawbacks of aggregate data and individual patient data meta-analysis.

**Table 1:** – Summary of advantages and drawbacks of aggregate data meta-analysis and individual patient data meta-analysis

	Advantages	Drawbacks
**Aggregate data meta-analysis**	Easier to perform	It is dependent on the original data quality and how the authors report it
	Require less time	Primary and secondary study endpoints may differ from those examined in the meta-analysis
	Less expensive	Negative findings are more likely to be overlooked due to publication bias
		Limited power to analyze treatment effects in specific subgroups of interest that differ from those evaluated in primary studies
**Individual patient data meta-analysis**	Preliminary and unpublished data can be included, reducing the risk of publication bias	Time-consuming due to data acquisition, extraction, analysis, and the need for collaboration
	Comprehensive assessment of the protocol, methods, and overall study quality	Data collection from certain studies, particularly the oldest, may be difficult
	Allows for subgroup analysis better to understand an intervention's impact at the patient level	More expensive
	If applicable, statistical analysis can be adjusted to baseline differences	More expertise required

## What are the differences between individual patient data and aggregate data meta-analysis from statins trials in terms of results?

The Cholesterol Treatment Trialists’ Collaboration, a meta-analysis of individual patient data with 170.000 patients and 26 clinical studies, aimed to assess the safety and efficacy of more intensive reductions of LDL-C.^2^Individual participant data from randomized clinical trials comparing more intensive versus less intensive statin regimens (5 trials; 39,612 individuals) and statins versus placebo were included (21 trials; 129,526 individuals). They showed a reduction in major cardiovascular outcomes for every 1 mmol/L reduction in LDL-C (rate ratio, 0.78; 95% confidence interval [CI], 0.76 to 0.80; p<0.0001).^2^

The aggregate data meta-analysis published by Byrne et al. to evaluate a similar question obtained inconclusive results.^4^The association between LDL-C reduction and myocardial infarction was not statistically significant, the association between LDL-C reduction and death was significant only in the relative but not in the absolute scale, and the association between LDL-C reduction and stroke was significant with a reduction in both absolute and relative risks.^3^This study used meta-regression, which analyzes the aggregate reduction in LDL-C and (aggregate) effect size from each trial. By contrast, in the individual participant data, meta-analysis considers the variation in LDL-C and outcome for each patient, with a much greater granularity of information.^3^

How one makes sense of these discrepant results? Furthermore, in what situations are aggregate data meta-analysis and an individual patient meta-analysis expected to generate identical answers for a specific question?

When the question concerns the comparative effect of two alternative treatments on a clinical outcome in a given population, the pooled estimate of the effect of either aggregate data or individual patient data meta-analysis should be similar.^5^

However, discrepancies may emerge for several reasons. First, there may be differences in the original studies enrolled: high-intensity statins regimens versus low-intensity regimens were considered only in the individual patient data meta-analysis. As a result, patients enrolled in the aggregate data meta-analysis were at lower cardiovascular risk. This characteristic of the LDL-C aggregated data meta-analysis help explain smaller absolute effect sizes, which are dependent on baseline risk (assuming constant relative risk reduction across different baseline risk).

Second, aggregate data meta-analysis has a lower power to detect relationships between patient-level characteristics and effect sizes. A meta-epidemiological study shows that estimates from meta-regression of aggregate data are less precise than those from individual patient data meta-analyses.^6^Random error on meta-regression estimates will often cause them to differ substantially from those obtained on individual patient data meta-analysis.^7^This fallacy is known as aggregation bias, which is defined by the assumption that an association between two group-level variables equals the association between the corresponding variable at the individual level.^7^

Third, not all characteristics that may significantly impact how an intervention works can be adequately assessed in an aggregate data meta-analysis, most commonly because effects according to the baseline characteristics of interest are not available in the reported papers.^7^

Fourth, in the case of the LDL-C meta-analyses, the individual patient data meta-analysis assessed the association between LDL-C variation and a composite outcome (coronary death or non-fatal myocardial infarction). In contrast, the aggregate data meta-analysis assessed the association between LDL-C and death, stroke, or myocardial infarction separately.^2 , 3^

As the number of events for a composite outcome is greater, the precision of effect estimates increases. When original studies did not report treatment effects for the combined outcome of interest, meta-analysis using aggregate data is not possible. Conversely, generating and analyzing combined outcomes is feasible by assessing original trial databases.

Therefore, compared to aggregate data meta-analyses, individual patient data meta-analyses are superior in investigating relationships between effects on intermediate variables and clinical outcomes and examining potential treatment modifiers. Conversely, a meta-analysis of aggregate or individual patient data should offer similar answers for questions of treatment effect, as long as they use the same definitions for population, treatment, comparator, and outcomes and include the same original studies.
